# Representative sampling of natural biofilms: influence of substratum type on the bacterial and fungal communities structure

**DOI:** 10.1186/s40064-016-2448-2

**Published:** 2016-06-21

**Authors:** Jennifer Hellal, Caroline Michel, Vanessa Barsotti, Valérie Laperche, Francis Garrido, Catherine Joulian

**Affiliations:** Département Eau Environnement Ecotechnologies, BRGM D3E/BGE, 3 Av. Claude Guillemin, BP. 36009, 45060 Orléans Cedex 2, France

**Keywords:** Natural biofilm, Sampling substrata, Sampling strategy, Freshwater, Bacteria, Fungi, DGGE

## Abstract

In situ biofilm sampling is a key step for the study of natural biofilms and using methodologies that reflect natural diversity is necessary to guarantee representative sampling. Here, we focalise on the impact of the type of substrata on which biofilms grow on bacterial and fungal communities’ structure. The indirect molecular approach, Denaturing Gel Gradient Electrophoresis (DGGE) of a gene fragment coding for either 16S rRNA or 28S rRNA, for bacteria or fungi respectively, was used to evaluate the variability of microbial community structures among different biofilm substrata: natural (pebbles, live plants, wood and sediment), or artificial (glass, Plexiglas^®^ and sterile wood), in a small river (the Loiret, France). Multivariate statistics, band richness and diversity indexes (Shannon and Simpson) were used to highlight variations in community structure between substrata. Results showed variations of bacterial and fungal diversity between different substrata according to substratum properties/origin (natural or artificial, organic or inorganic) but there was no optimal substratum for sampling, and artificial substrata were not significantly less applicable than natural substrata. Pooling 4 different substrata types allowed a higher bacterial and fungal biodiversity recovery. Point contact sampling may thus gain in robustness by increasing the number of substrata considered. Fungal species richness was similar to the bacterial one on most substrata which suggested they should be more frequently considered in riverine biofilm studies.

## Background

One of the main difficulties in studying natural aquatic biofilms is the quasi impossibility of growing them in identical conditions ex situ without modifying them, or artificially selecting a number of species. Indeed, many factors can influence biofilm development such as substratum roughness, charge, hydrophobicity, critical substratum tension, wettability, microtopography, organic and mineral content and shear current (Anderson-Glenna et al. 2008; Battin et al. 2003). In order to avoid these biases, most studies on natural aquatic biofilms are carried out in situ, generally by sampling pebbles (*i.e*. point contact method) (Anderson-Glenna et al. 2008; Lear et al. 2008; Lyautey et al. 2003, 2005) or by immerging artificial substrata, generally glass but also tiles, Plexiglas^®^ or wooden planks, and harvesting them once a biofilm has developed (Kralj et al. 2006; Kröpfl et al. 2006; Tien et al. 2009).

A few studies have previously sought to test the robustness of artificial substrata for the growing and sampling of algal biofilms, microbial biofilms and other organisms such as diatoms. On algal communities, Cattaneo and Amireault (1992) showed, in a survey of 27 separate studies, that artificial substrata can misrepresent the quantity and quality of natural algal communities. Branco et al. (2010) also studied the effects of artificial substrata on algal development and showed that physical structure of the substratum did not affect species richness but did alter the abundance of the macroalgal community. A third example is the work carried out by Danilov and Ekelund (2001). These authors compared three types of substratum, glass, wood and plastic for sampling periphyton in lakes and showed that glass supported the highest biomass compared to wood whereas no algae grew on the plastics used; instead of algae these were covered in a layer of bacterial slime. Diatoms on the other hand have been grown efficiently on artificial substrata for nearly 100 years without having any effect on their abundance and diversity (Lane et al. 2003). Concerning bacterial communities, there are very few studies measuring the impact of artificial substrata on bacterial biofilms although, as we mentioned above, they are extensively used for collecting samples (Kröpfl et al. 2006; Möhlenhoff et al. 2001; Danilov and Ekelund 2001; Rozej et al. 2015). Lyautey et al. (2003) suggested that natural epilithic biofilms presented a higher diversity than those grown on artificial substrata in rivers. Kröpfl et al. (2006) compared algae and bacterial development on several natural and artificial substrata in a Hungarian river (granite, andesite, Plexiglas^®^ and polycarbonate) and recommended Plexiglas^®^ as the more efficient. On fungal communities, to our knowledge there are no existing studies that have evaluated fungal colonisation of artificial substrata. Moreover, there are very few existing studies on the genetic structure of fungal communities in riverine biofilms, the two most recent are Fischer et al. (2009), who studied fungal diversity on fallen leaves and Das et al. (2008) who also focused on leaves and compared fungal diversity on two species. However several studies have underlined the importance of fungal communities in riverine biofilms (Baldy et al. 1995; Gessner and Chauvet 1994; Golladay and Sinsabaugh 1991).

The aim of the present study was to evaluate the variability of bacterial and fungal diversity amongst natural and artificial substrata and thus to contribute to optimising microbial biofilm sampling in aquatic ecosystems. We based our approach on a fingerprinting tool: the culture-independent method DGGE of amplified fragments of the genes coding for 16S (bacteria) or 28S (fungi) rRNA. We thus considered the following two aspects of biofilm sampling that are susceptible to create heterogeneity: (i) the variability of microbial community structure between and within natural substrata types and (ii) the efficiency of artificial substrata (glass, Plexiglas^®^ and wood) for growing natural bacterial and fungal biofilms. DGGE data were analysed using multivariate statistics, diversity indexes and species richness.

## Methods

### Study site and sampling

Biofilms and water samples were collected from the edge of the river Loiret (13 km long, average speed 1 m/s) that flows into the Loire in central France (47°51′48″N and 1°48′26″E). At this point the river is between 20 and 25 m wide and no more than 1 m deep in its centre. The banks are shaded by trees (mainly popular, lime and chestnut) and many macrophytes grow all across the river demonstrating good light penetration. Three sampling campaigns were carried out. In June 2009, natural biofilms were collected from four abundant substrata: live plants, wood (fallen tree bark), pebbles, and sediment substratum. At the same time, sterile artificial substrata (autoclaved 121 °C, 1 h) were immerged in the river (glass, Plexiglas^®^ or wooden slices made from a chestnut log) and then collected 5 weeks later during the second campaign in August (Tien et al. 2009). A third campaign was carried out in November 2009 for additional sampling of natural substrata.

The samples were kept in sterile plastic bags at 4 °C. The same day as collection the samples were crushed in sterile NaCl 0.7 % (plants, wood), brushed with sterile bottle cleaners and again suspended in NaCl 0.7 % (pebbles, glass, Plexiglas^®^) or directly suspended in NaCl 0.7 % (sediment).

### DNA extraction

One ml of each suspension was centrifuged at 16,000*g* for 5 min and DNA extractions were carried out on the pellets using the FastDNA^®^ SPIN kit for Soil (MP Biomedicals, France).

### PCR

The variable region V3–V5 of the gene coding for 16S rRNA was amplified according to Muyzer et al. (1993) using the bacteria specific primers 341F (5′-CTA CGG GAG GCA GCA G-3′) with a GC clamp at its 5′ end (5′-CGC CCG CCG CGC GCG GCG GGC GGG GCG GGG GCA CGG GGG GC G-3′) and 907R (5′-CCG TCA ATT CCT TTG AGT TT-3′), and the following programme: 95 °C for 2 min followed by 32 cycles of 95 °C for 30″, 55 °C for 30″ and 72 °C for 45″ followed by 5 min final elongation at 72 °C. The gene coding for 28S rRNA was amplified using previously published primers 403f (5′-GAC TCC TTG GTC CGT GTT-3′) and 662r (5′-GTG AAA TTG TTG AAA GGG AA-3′) with a GC clamp at the 5′ end of primer 662r (5′-CGC CCG CCG CGC GCG GCG GGC GGG GCG GGG GCA CGG GGG GC G-3′), corresponding to the coordinates 403–422 and 645–662 of the reference *Saccharomyces cerevisiae* 28S rRNA gene, respectively (Sandhu et al. 1995). These primers have been successfully used for DGGE in a previous study by Möhlenhoff et al. (2001) and Xin-Yu et al. (2010). The following programme was used (Sandhu et al. 1995): 32 cycles, carried out at 94 °C for 30″, 50 °C for 1 min and 72 °C for 2 min. PCR was performed in an I-Cycler (Biorad, France) and PCR products were quantified by agarose gel electrophoresis using Smart Ladder^®^ (Invitrogen).

### DGGE

DGGE was carried out in a vertical D-code system (Biorad) as described by Muyzer et al. (1993). Denaturation gradient of the 8 % acrylamide gels ranged from 40 to 60 % (urea/formamide) for both 16S and 28S rRNA gene amplicons. The migrations ran for 17 h at 80 V in a 1 % TAE buffer heated to 60 °C. Gels were stained with ethidium bromide and digital images were taken and analysed with the Gel-doc/Quantity One exploitation system (Biorad). The software Quantity One (Biorad) was used to compare DGGE profiles on the gel photo.

### Data analysis

Two types of data matrices were created from DGGE gels: qualitative binary matrices taking into account presence/absence of each band (0: absence, 1: presence) and quantitative matrices of the relative intensity of each band in relation to the total profile intensity.

All statistical analyses were carried out with XLSTAT Version 2014.2.01.

The binary matrices (presence/absence) of DGGE bands created for bacterial and fungal communities were analysed using multi correspondence analysis to compare artificial versus natural substrata or construction of a similarity matrix based on the Jaccard index and graphically displaying the results as a dendrogram to highlight variations in community structure within and between natural substrata.

The quantitative matrices of relative band intensity obtained from the DGGE analysis were analysed using principal component analysis to visualise variability between bacterial and fungi communities on triplicates of natural substrata.

According to Hill et al. (2003), the relative intensities of each DGGE band for all the samples were used to calculate the Shannon (H′) and the Simpson’s (D or 1/D) indexes.

Shannon’s index (H′) quantifies the heterogeneity of the studied system (i.e. a microbial community in our case) and ranges between 0 and Hmax. H′ is minimal if all the individuals of a community belong to the same species or if in a community all the present species are represented by one individual except for one species which is represented by the remaining individuals of the community. H′ is maximal when the individuals are equally distributed among the species.

Simpson’s index (D or 1/D as in this paper) measures the probability that two individuals picked at random do not belong to the same species. Diversity is low if 1/D is low and vice versa. Differences in diversity indexes between biofilm samples according to their substrata were assessed using one-way ANOVA or a Fisher test with a 5 % threshold. Samples showing a significant substrata effect were then tested using a Tukey test.

## Results

### Impact of natural versus artificial substrata on microbial community structures

Diversity indexes were calculated in order to compare the structures of the microbial communities in the biofilms. Table 1 gives the Richness, Shannon’s index (H′ and H′max) and Simpson’s index (1/D) for samples of biofilm in comparison to the surrounding river water.

**Table 1:** Band richness and diversity indexes calculated from the 16S and 28S rRNA DGGE profiles using relative band intensities (% of band intensity in relation to total profile intensity) for the natural biofilm substrata (Nat) in comparison to the artificial biofilm substrata (Art)

Bacteria (16S)					Fungi (28S)			
Biofilm surface	Band richness	Shannon index (H′)	H′ max	Simpson index (1/D)	Band richness	Shannon index (H′)	H′ max	Simpson index (1/D)
Nat wood	15	2.7	2.7	14.3	19	2.9	2.9	16.3
Nat plant	14	2.6	2.6	11.7	24	3.2	3.1	20.7
Nat pebble	15	2.7	2.7	14.0	26	3.3	3.2	23.5
Nat sediment	19	2.9	2.9	17.5	25	3.0	3.2	18.3
Art Plexiglas^®^	12	2.3	2.4	13.3	29	3.3	3.3	23.0
Art wood	19	2.9	2.9	16.7	30	3.3	3.4	21.9
Art glass	10	1.1	2.3	34.3	24	3.1	3.1	20.8
River water	11	2.4	2.3	11.0	33	3.1	3.4	20.4

The number of bands in the 16S rRNA DGGE ranged between 10 and 19 bands, the highest numbers being found in the natural sediment and artificial wood samples (Table 1). The band numbers found in the 28S rRNA DGGE profiles were considerably higher, ranging from 19 to 33 with the highest numbers in artificial wood and Plexiglas^®^ samples and the water sample.

Shannon’s index (H′: heterogeneity of the studied system) varied little between samples and was overall high for all samples, and generally very close to H′max, suggesting a high heterogeneity in each biofilm. The only exception was the biofilm recovered from the glass substratum where H′ was less than half H′max. Simpson’s index (1/D: probability that two individuals picked at random do not belong to the same species) ranged between 10.9 and 34.2, and between 16.3 and 23.5 for bacteria and fungi, respectively. The lowest diversity was found for bacteria on the river glass sample whereas results were similar for fungi on artificial and natural substrata. Differences in diversity indexes between natural and artificial substrata were not overall significant (Fisher Test p value >0.05) except for the bacterial Simpson index (1/D) which differed, probably induced by the artificial glass substrata which had a high Simpson index of 34.2.

**Fig. 1:**
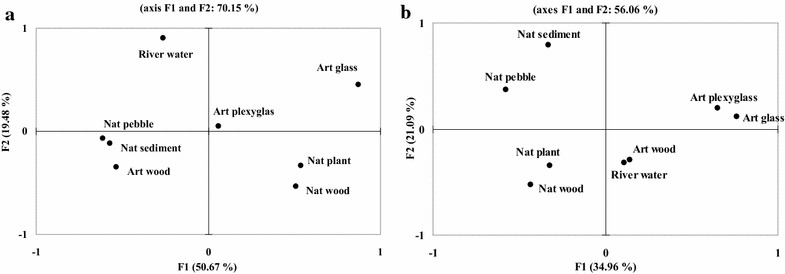
Multiple correspondence analysis of the presence/absence of DGGE bands in the biofilms of natural (Nat) and artificial (Art) substrata [**a** bacteria (16S rRNA), and **b** fungi (28S rRNA)]

The projections established by multiple correspondence analyses of the biofilm DGGE presence/absence profiles (Fig. 1) differed when considering 28S rRNA or 16S rRNA gene amplicons. When considering fungal communities (Fig. 1b), the community structure separated the biofilm substrata into natural versus artificial ones along F1 and organic versus inorganic ones along F2. River water was closest to the community developed on artificial wood. Concerning 16S rRNA gene profiles, the substrata formed 3 clusters plus the river water, these being artificial inorganic substrata (Art glass and Art plexyglas), natural organic substrata (Nat plant and Nat wood), and natural inorganic substrata (Nat pebble and Nat sediment) plus the artificial organic substratum (Art wood) (Fig. 1a).

### Variability of microbial community structure within and between natural substrata

**Fig. 2:**
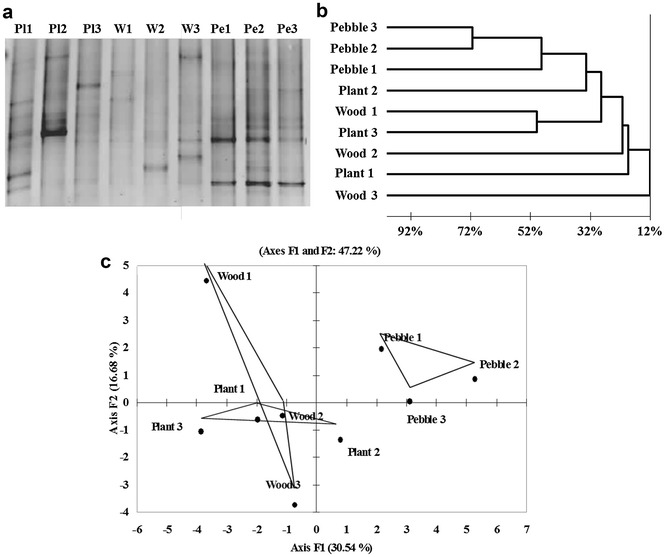
Variability of the bacterial community structure within substrata types for biofilms collected in November 2009 from replicates of different natural substrates: **a** Negative image of the DGGE gel, **b** Dendrogram of Jaccard distances calculated from the DGGE band presence/absence matrix, and **c** Principal component analysis of band intensity profiles of gene fragments coding for 16S rRNA (only the first two factors/components have been represented)

**Fig. 3:**
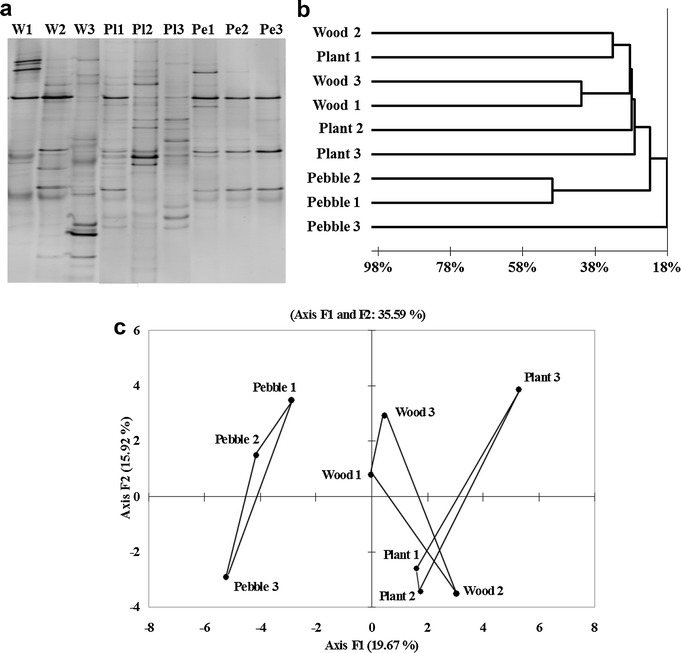
Variability of the fungal community structure within substrata types for biofilms collected in November 2009 from replicates of different natural substrates: **a** Negative image of the DGGE gel, **b** Dendrogram of Jaccard distances calculated from the DGGE band presence/absence matrix and **c** Principal component analysis of band intensity profiles of gene fragments coding for 28S rRNA (only the first two factors/components have been represented)

The variability of the microbial community structure within and between substrata types was tested for natural substrata using the triplicate samples of different biofilm substrata collected in November 2009 (wood, plants and pebbles). The DGGE gels obtained are shown in Figs. 2a and 3a.

Based on the DGGE analysis, diversity indexes were calculated in order to compare the structures of the microbial communities for each triplicate substratum (Table 2). When considering bacterial communities, the number of bands in the 16S rRNA DGGE, Shannon’s index, H′max and Simpson’s index were not significantly different (ANOVA, p value >0.05). This was not the case for the fungal communities where differences were observed between substrata, mainly between the organic ones (plant and wood) and the inorganic pebbles for which band richness, the Shannon index and H′max were significantly different (ANOVA and Tukey test, p value <0.05). The Simpson index on the other hand highlighted a significant difference in the level of diversity between the plant and wood substrata but not between the latter and the pebbles (ANOVA and Tukey test, p value <0.05).

**Table 2:** Band richness and diversity indexes calculated from the 16S and 28S rRNA DGGE profiles using relative band intensities (% of band intensity in relation to total profile intensity) for the natural biofilm substrata in November 2009

Bacteria (16S)					Fungi (28S)			
Biofilm surface	Band richness	Shannon index (H′)	H′ max	Simpson index (1/D)	Band richness	Shannon index (H′)	H′ max	Simpson index (1/D)
Plant 1	8	1.9	2.0	8.6	24	3.2	3.1	21.5
Plant 2	7	1.7	1.9	4.7	27	3.1	3.2	28.8
Plant 3	11	1.9	2.3	8.3	27	3.3	3.2	25.3
Wood 1	7	1.6	1.9	4.6	22	3.0	3.0	16.9
Wood 2	8	1.9	2.0	6.6	22	3.0	3.0	17.9
Wood 3	8	1.6	2.0	8.2	24	3.2	3.1	22.3
Pebble 1	11	1.7	2.3	13.1	17	2.7	2.8	13.0
Pebble 2	13	2.1	2.5	8.9	18	2.9	2.8	15.6
Pebble 3	11	2.1	2.3	9.9	13	2.5	2.5	10.3
River water	15	2.6	2.7	13.9	25	3.3	3.2	23.5

A similarity matrix was generated from the binary presence/absence data obtained from the DGGE using the Jaccard index and the results are displayed graphically in Figs. 2b and 3b for bacterial and fungal communities respectively. Dendrograms both for bacterial and fungal communities highlight more similarities between the communities found in the biofilms formed on pebbles whereas plant and wood-biofilm structures differed. This reflects the visual observation of the DGGE gels (Figs. 2a, 3a) where pebble profiles can be seen to be similar.

This was also demonstrated through the further analysis of the bacterial and fungal community structures that were assessed with a principal component analysis (PCA) applied to the relative intensity matrices (Figs. 2c, 3c).

The two principal components of the PCA are presented in Fig. 2c (16S rRNA gene) and Fig. 3c (28S rRNA gene). Whereas the DGGE profiles from pebbles formed tight clusters in both the 16S rRNA gene and 28S rRNA gene analysis, the biofilm diversity profiles issue from plants and wood were on the one hand more dispersed, indicating variations in their composition on identical substrata, and on the other hand more entwined than for the pebble profiles, indicating that the communities in biofilms formed on bark and plant substrata were more similar to each other than those formed on pebbles.

### Sampling strategy to account for biodiversity

**Fig. 4:**
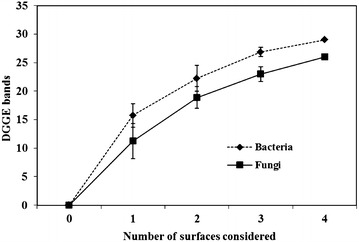
Increase in the number of DGGE bands (16S rRNA and 18S rRNA) depending on the number of biofilm substrata considered. The standard deviation is that of the eight random combinations for 1, 2, 3 or 4 substrata

To estimate the minimum number of biofilm substrata to be taken into account for a reliable diversity estimation when sampling aquatic biofilms, we calculated the increase in new DGGE bands induced by taking into account 1, 2, 3 or 4 different biofilm substrata when considering either 16S or 28S rRNA gene (Fig. 4). The curve trends were similar for bacteria and fungi although overall we found around 30 different bands of 16S rRNA and only 25 of 28S rRNA. Globally the increase in new bands decreased the more biofilm substrata were considered, pointing towards a maximum diversity for 4 substrata and more.

## Discussion

Many biotic and abiotic factors such as season and river features (canopy coverage, water height, current velocity, water temperature, etc.) influence the community structure and composition of natural biofilm that develop in rivers. The substratum itself can also impact biofilm development and biodiversity. As biofilm sampling is a key step for biofilm studies, substratum impact has to be taken into account. The objective of this work was to evaluate the impact of the substratum type on biofilm diversity by focusing on bacterial and fungal communities.

### Methodological aspects

Lyautey et al. (2005) extensively discussed the use of the 16S rRNA PCR-DGGE approach for studying riverine biofilms, using the same primers (341F-GC and 907R) as the present study. Although this approach has limitations [for example co-migration of PCR fragments from different species in the same DGGE band, formation of multiple bands during amplification of genes from a single genome or detection of artificial bands when analyzing complex DNA templates (Fromin et al. 2002; Lyautey et al. 2005; Piterina and Pembroke 2013)], and requires methodological settings and a standardised procedure, Lyautey et al. (2005) concluded that this method remains a powerful tool for investigating biofilm bacteria and better understanding biological processes in riverine biofilms.

Fungal community analysis using 28S rRNA PCR-DGGE has however hardly been used to evaluate fungal diversity; twice in soils (Diedhiou 2007; Xin-Yu et al. 2010) and once in painted art work (Möhlenhoff et al. 2001). The authors that designed the original primers (Sandhu et al. 1995) checked their potential to amplify 23S rRNA gene sequences from bacteria by searching amongst 539 bacterial sequences on Gene bank (http://www.ncbi.nlm.nih.gov/genbank/). No suitable annealing sites were found and under stringent conditions these two primers appeared incapable of amplifying 23S rRNA gene. Since 1996, the DNA databases have considerably increased so we checked the specificity of the primers on Probe Match (Ribosomal Database Project http://rdp.cme.msu.edu/probematch/search.jsp). The result yielded no match with bacterial DNA which reinforces the specificity of these universal fungal probes.

### Bacterial and fungal diversity

Overall the number of different DGGE bands we identified for bacteria and fungi is comparable with previous results (Lyautey et al. 2003; Araya et al. 2003) as are the results obtained for richness and diversity indexes for bacteria (Lyautey et al. 2003). The various natural and artificial substrata we have used in this study can thus be used to study the impact of substratum type on microbial diversity and investigate representative sampling of natural biofilms.

Results showed that the average number of bands found on biofilm DGGE profiles was higher for fungi. This gives interesting perspectives for phylogenetic studies of this group of organisms which is rarely focused on when considering biofilm diversity even if several authors have previously demonstrated fungi abundance in aquatic biofilms and their implication in degrading organic matter in streams and rivers (Golladay and Sinsabaugh 1991).

### Microbial biodiversity on natural surfaces

Although the influence of biofilm substrata has previously been investigated, the studies which have been done so far have used cell abundance, metabolic activities (respiration) or biomass approaches to determine biofilm variability (Cattaneo and Amireault 1992; Danilov and Ekelund 2001; Golladay and Sinsabaugh 1991; Kralj et al. 2006; Kröpfl et al. 2006; Tien et al. 2009) but not molecular fingerprinting methods such as DGGE. In the present study, this technique enabled us to reveal bacterial and fungal biofilm structure among several natural and artificial substrata and highlight several differences. Analyses of DGGE profiles using multivariate statistics enabled us to highlight differences in microbial community structure between the different natural biofilm surfaces from the same sampling spot. Notably, these analyses showed that microbial communities growing in biofilms on organic substrata (plants and wood) were significantly more similar to each other than to those growing on inorganic pebbles. These observations could possibly be linked to the organisms’ metabolisms, heterotrophic ones developing where they can find accessible nutrients and autotrophic bacteria meeting less competition on mineral surfaces such as pebbles. For fungi we also observed less DGGE bands in biofilms from pebbles (inorganic substratum) which joins this hypothesis. Biofilm sampling in aquatic systems would thus gain in sampling several dominant organic and inorganic substrata. This is supported by our results which showed that maximum diversity was obtained by sampling 3–4 different substrata (Fig. 4). This approach could be compared to sampling strategies used in ecology such as the principle of the species-area curve (Preston 1962). Sampling should also consider replicates as previously emphasised. Indeed, statistical analyses not only revealed differences between different substrata but also highlighted differences amongst similar substrata sampled in the same spot where community structure differed especially between the organic substratum (plants and wood) whereas pebble associated biofilms varied less.

### Microbial diversity on artificial substrata

This study also aimed to compare bacterial and fungal development on artificial substrata and thus evaluate their efficiency for growing natural biofilms. Indeed, if we can demonstrate that the diversity and species richness found on artificial substrata is representative of the diversity found on natural substrata then it reinforces their use for studying riverine biofilms. The results first underlined structural differences between biofilms developed onto artificial or natural wood potentially due to wood treatment (sterilisation) and/or in situ incubation time. The DGGE profiles established for fungi separated, on the one hand, the natural substrata from the artificial ones, and on the other hand, the organic ones from the inorganic ones. The results obtained for bacteria profiles differed from that for fungi and it was very difficult to identify trends based on the presence/absence of DGGE bands. For bacteria, the lowest diversity was found on glass samples suggesting that this substratum widely used for in situ biofilm sampling, is possibly not the best one. However, as a whole, no significant differences were observed for the diversity indexes between artificial and natural substrata, thus reinforcing their potential use for growing and sampling biofilms in riverine water.

## Conclusion

An increasing number of studies are carried out on aquatic riverine biofilms and robust sampling methods are thus necessary to fully appreciate microbial biofilm diversity in in situ studies. Glass slides are widely used for growing riverine biofilms as is the sampling method by point contact involving pebbles. However, to our knowledge, no studies have investigated the effect of biofilm substratum (natural or artificial) on the genetic structures of biofilm microbial communities in such complex ecosystems, and thus on sampling representativeness. Moreover, only few studies have focused on fungal community fingerprints in such ecosystems although several authors have underlined their importance, especially on organic substrata such as leaves or wood.

The results obtained in the present study reveal the variations of bacterial and fungal diversity between different substrata, bringing us to suggest that point contact sampling may gain in robustness by increasing the different substrata considered. Our results also highlight a high fungal diversity on most substrata which had not been investigated previously using a fingerprinting approach, and which can evolve differently from bacterial diversity. Bacterial diversity alone is thus not representative of the global microbial diversity in riverine biofilms, and this study underlines the necessity to take into account fungal diversity as well as the bacterial one. All these results thus emphasize that (i) there is no optimal substratum for sampling, (ii) artificial substrata are not significantly less applicable than natural substratum, (iii) the necessity to sample different substrata, consisting of a mix of at least 3–4 organic and inorganic substrata, and (iv) the necessity to analyse both bacterial and fungal communities. Concerning this last point, although DGGE has proved to be a good community screening tool it could now be completed by a metagenomics analysis that would provide additional insights to better characterise fungal and bacterial diversities and functions in riverine biofilms.
